# How have trends in lifespan variation changed since 1950? A comparative study of 17 Western European countries

**DOI:** 10.1093/eurpub/ckv185

**Published:** 2015-11-27

**Authors:** R. Seaman, A. H. Leyland, F. Popham

**Affiliations:** 1 MRC/CSO Social and Public Health Sciences Unit, University of Glasgow, 200, Renfield Street, Glasgow G2 3QB, UK

## Abstract

Lifespan variation adds to life expectancy by measuring the inequality surrounding age of death that a population faces. Countries that tackle premature mortality generally have decreasing lifespan variation but this is the first study to compare and statistically assess when and to what extent trends in lifespan variation have changed across Western Europe. Lifespan variation was measured using e† and joinpoint regression analysed the timing and rate of change. Trends have been mostly downward with the recent exception of men in Scotland, Northern Ireland, Ireland and Finland where trends have flattened or show slight increases. Future research aimed at identifying the ages and causes of death, driving trends in these countries, is key to preventing increasing inequalities.

## Introduction

Research has established life expectancy and mortality differences between Western European countries^1^^,^^2^ and has now started to explore lifespan variation differences.^3^^,^^4^ Lifespan variation adds to life expectancy by summarising the inequality in age of death. Decreasing lifespan variation suggests decreasing uncertainty surrounding average lifespan as deaths are compressed around a common age. This is important as lifespan variation may not change even if life expectancy is increasing. For example, if a population has reduced mortality at older ages faster than it has reduced premature mortality it will have achieved increases in life expectancy but not mortality compression.^5^ Therefore, measuring life expectancy and lifespan variation helps to demonstrate the extent to which improvements in average population health and reductions in mortality inequality have been achieved simultaneously.^5^^,^^6^

The Western European countries which have achieved the highest life expectancy and lowest lifespan variation are generally those which have tackled premature mortality. Scotland^4^ and Finland^6^ are two countries where lifespan variation may have changed to be stagnating or even increasing despite improvements in life expectancy. However, it is not clear whether these changes in trends for lifespan variation are significant or when they began exactly. It is also not clear if similar changes in trends for lifespan variation have been seen in other Western European countries. These are important research questions to answer for two reasons, firstly to identify countries that are failing to reduce inequalities to the same extent as comparable countries, and secondly to identify when any changes in trends may have started in order to inform future studies into the causes of mortality inequalities. Using trend analysis techniques we explore trends in lifespan variation in 17 Western European countries from 1950 onwards.

## Methods

The Human Mortality Database (HMD) provides annual, sex specific lifetables for individual countries from which we calculated lifespan variation. Seventeen Western European countries that had data available and which have been used in existing research were identified.^2^ We used lifetables from 1950 to 2011, although some countries had data only from the mid-1950s (see Supplementary table for exact years).

Several measures of lifespan variation exist and are highly correlated (technical summary available elsewhere^7^). We chose e† as it covers the whole age range. It is interpreted as the average number of years of life lost per death, giving it an intuitive meaning. It is calculated by summing remaining life expectancy at each age weighted by the proportion of deaths at that age, and was calculated for each year, sex and country using Stata SE13.

Once *e*† was calculated the data were imported into the joinpoint regression programme 4.0.4.^8^ Joinpoint regression identifies and quantifies changes to trends, and calculates the time points at which a change in trend is statistically significant by testing whether a multi-segmented line is a significantly better fit than a straight or less-segmented line. It is used to evaluate both the time point and the level of change across a time series for any given health outcome. Detailed information on the joinpoint regression programme is given elsewhere.^8^ We modelled the log of lifespan variation in order to calculate annual percentage change (APC).

## Results

**Figure 1:**
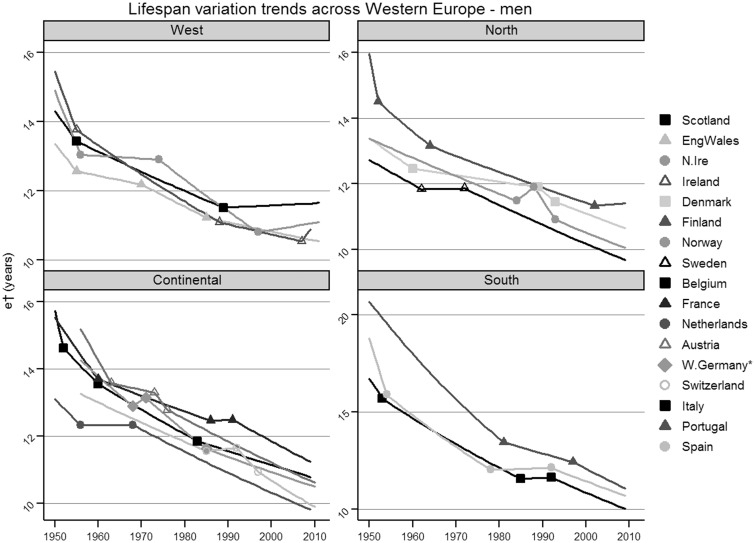
Results were produced using data obtained from the HMD (www.mortality.org/). *West Germany is the area of unified Germany formerly known as the Federal Republic of Germany (FRG). The territory has changed overtime but the statistics here represent the regions that made up the FRG

Figure 1 shows the modelled trends for lifespan variation for men in all 17 Western European countries, grouped by geographic area. The equivalent graph for women is provided as a Supplementary figure.

### Men

Since 1950 there has been a general decreasing trend for lifespan variation amongst men in Western Europe with some increases for short periods before returning to a downward trend. Rapid decreases in trends were found in the years immediately after 1950 with the steepest decline of −4.1% (95% CI −5.0 to −3.2) found in Spain between 1950 and 1954. However more recently, Scotland, Northern Ireland, Ireland and Finland have changed to a flattening or slightly increasing trend that has yet to be reversed.

Acknowledging the sometimes wide confidence intervals, Scotland has been experiencing a slightly increasing trend since 1989 (95% CI 1986–1993) with an APC of 0.1% (−0.0 to 0.1). Finland has had a slight increasing trend since 2002 (1981–2007) with an APC 0.1% (−0.4 to 0.5), Northern Ireland since 1997 (1992–2001) with an APC 0.2% (0.0–0.4%) and Ireland since 2007 (1983–2007) with an APC 1.6% (−1.5 to 4.7).

### Women

For women, all countries show a general decreasing trend since 1950, with some short periods of increasing lifespan variation, before returning to a decreasing trend. A similar rapid decline in lifespan variation is found for women, as was found for men, in the years immediately after 1950 with Spain again having the steepest decrease of −4.5% (−5.3 to −3.7%) between 1950 and 1954.

Lifespan variation shows a strong converging trend for women in Western Europe, not evident for men. However, Scotland is an exception as it has had higher levels of lifespan variation and a greater slowing in the rate of change, slowing from −1.9% (−2.3 to −1.6) between 1950 and 1956 to −0.1% (−0.3 to 0.2) now. This is in contrast to its nearest neighbour, England and Wales which has experienced an APC decrease of −1.3% (−1.6 to −0.9) between 1950 and 1956 to an APC decrease of −0.4% (−0.4 to −0.4) between 1970 and 2011. Scotland has had the highest level of lifespan variation for 22 of the past 59 years, and is the only country to still have a lifespan variation of over 10 years amongst women (10.4 years in 2011).

From the modelled data women in Sweden, The Netherlands, Norway, Finland, Spain and Switzerland have all had the lowest level of lifespan variation, and thus occupied the most favourable position, at more than one time point between 1950 and 2009. This finding is in contrast to men, for whom Sweden has consistently had the lowest lifespan variation.

## Discussion

This study adds to the growing body of research that is concerned with the timing and extent of change in trends for population health and mortality inequalities across Western Europe. It is the first to formally identify, quantify and compare changes for trends in lifespan variation, a novel measure of mortality inequality.

Most countries have been able to sustain a decreasing trend; however, lifespan variation for men in Scotland, Finland, Northern Ireland and Ireland has diverged to a flattening or slight increasing trend, with change occurring in recent, but different, years. The trends identified for Scotland and Finland are consistent with existing research which identified rising lifespan variation for the whole population in Scotland and across all social classes, except the highest, in Finland.^4^^,^^6^ For women across Western European countries, a strong converging trend is found, with the exception of Scotland where the level of lifespan variation has been higher and the rate of change has slowed.

These trends may be partly explained by continuing high levels of premature working age mortality, particularly amongst those socioeconomically deprived.^6^ Evidence for Scotland demonstrates high rates of premature deaths caused by violence, suicides and alcohol and substance abuse which are associated with rising socioeconomic inequality in mortality between 1981 and 2001, and Scotland’s slow improvements in life expectancy.^1^^,^^9^ In Finland, external causes of death amongst working age adults partly explained diverging trends in lifespan variation by social class.^6^ The contribution premature mortality has made to changes in lifespan variation in Scotland has not yet been estimated.

Future research could apply decomposition analysis to calculate the amount each age and cause of death contributed to the change in lifespan variation.^6^ This may identify the drivers of health gaps and gives an indication as to which public health actions should be taken to reduce them.

## Supplementary data

Supplementary data are available at *EURPUB* online.

## Funding

R.S. is funded by a UK Medical Research Council (MRC) 1+3 PhD studentship. A.L. is funded by the MRC (MC_UU_12017/5) and the Chief Scientist Office (SPHSU2). F.P. is funded by the MRC (MC_UU_12017/7).

*Conflicts of interest*: None declared.

Key points

- Lifespan variation, the amount of inequality surrounding age at death, is generally found to be lower in the countries with higher life expectancy and that have been more successful at reducing premature mortality.
- Trends for lifespan variation in some Western European countries have been studied but changes to trends have not been statistically assed and compared, this research aims to fill this gap and analyse changes to trends since 1950.
- Most countries in Western Europe have sustained a decreasing trend in lifespan variation with the exception of men in Scotland, Finland, Northern Ireland and Ireland that have diverged towards increasing inequality, but at different time points.
